# GLP-1(7–36) protected against oxidative damage and neuronal apoptosis in the hippocampal CA region after traumatic brain injury by regulating ERK5/CREB

**DOI:** 10.1007/s11033-024-09244-8

**Published:** 2024-02-19

**Authors:** Shuwei Wang, Aijun Liu, Chaopeng Xu, Jingxuan Hou, Jun Hong

**Affiliations:** Department of Neurosurgery, Tangshan Gongren Hospital, Tangshan, 063000 Hebei China

**Keywords:** Glucagon-like peptide-1, Traumatic brain injury, Oxidative stress, Apoptosis, Hippocampal CA fields

## Abstract

**Background:**

Glucagon-like peptide-1 (GLP-1) (7–36) amide, an endogenous active form of GLP-1, has been shown to modulate oxidative stress and neuronal cell survival in various neurological diseases.

**Objective:**

This study investigated the potential effects of GLP-1(7–36) on oxidative stress and apoptosis in neuronal cells following traumatic brain injury (TBI) and explored the underlying mechanisms.

**Methods:**

Traumatic brain injury (TBI) models were established in male SD rats for in vivo experiments. The extent of cerebral oedema was assessed using wet-to-dry weight ratios following GLP-1(7–36) intervention. Neurological dysfunction and cognitive impairment were evaluated through behavioural experiments. Histopathological changes in the brain were observed using haematoxylin and eosin staining. Oxidative stress levels in hippocampal tissues were measured. TUNEL staining and Western blotting were employed to examine cell apoptosis. In vitro experiments evaluated the extent of oxidative stress and neural apoptosis following ERK5 phosphorylation activation. Immunofluorescence colocalization of p-ERK5 and NeuN was analysed using immunofluorescence cytochemistry.

**Results:**

Rats with TBI exhibited neurological deterioration, increased oxidative stress, and enhanced apoptosis, which were ameliorated by GLP-1(7–36) treatment. Notably, GLP-1(7–36) induced ERK5 phosphorylation in TBI rats. However, upon ERK5 inhibition, oxidative stress and neuronal apoptosis levels were elevated, even in the presence of GLP-1(7–36).

**Conclusion:**

In summary, this study suggested that GLP-1(7–36) suppressed oxidative damage and neuronal apoptosis after TBI by activating ERK5/CREB.

## Introduction

Traumatic brain injury (TBI) is a globally recognized health disaster. The high morbidity and mortality associated with TBI significantly increase the global economic burden [1–4]. During TBI, some neurons are directly mechanically damaged, but many others die due to secondary injury [5, 6]. As the cascade reactions progress after TBI, a series of pathophysiological responses (e.g., neural survival, oxidative stress, neuroinflammation and autophagic response) leads to tissue injury and cellular damage [6–8]. After TBI, the released reactive oxygen species (ROS) can significantly influence lesion size, trauma severity, and disease progression [9, 10]. Prior studies have suggested that oxidative stress plays a pivotal role in neuroinflammation and neural cell survival [11, 12]. Therefore, targeting oxidative stress may be a promising therapeutic option for improving the prognosis of TBI [13].

Recent studies have revealed that glucagon-like peptide-1 (GLP-1) exerts significant neuroprotective effects in a wide variety of central nervous system diseases, including ischaemic stroke and neurodegenerative disorders [14–16]. . GLP-1 exists in the circulation mainly as GLP-1(7–36) amide and GLP-1(7–37) [17]. Among them, GLP-1(7–36) amide is the most abundant [18]. A previous study showed that GLP-1 (7–36) possessed a neuroprotective property in Alzheimer’s Disease (AD) by targeting multiple physiological pathways [19, 20]. Evidence has shown that GLP-1 is capable of protecting neuronal cells in vitro from glutamate-induced oxidative stress [21]. Furthermore, GLP-1 plays an important role in the inhibition of excitotoxic neuronal death in cultured neurons [22]. This has led us to hypothesize that GLP-1(7–36) may also protect against secondary injury through multiple pathways, consequently improving neurological functions post-TBI, including inhibition of oxidative stress and apoptosis.

## Materials and methods

### TBI model and group

All animal experiments followed the National Institutes of Health Guide for the Care and Use of Laboratory Animals [23]. The study was approved under ethics by the Ethics Committee of Tangshan Gongren Hospital (Approval No. GRYY-LL-2020-100). Sixty male Sprague‒Dawley rats (10–12 weeks old) weighing approximately 250 g were chosen for the experiment. Rats were provided with ad libitum access to water and food. All rats were housed under optimal environmental conditions of 22 °C ± 2 °C and a 12-h light/12-h dark cycle. The TBI model is based on previously published models [24, 25]. Forty-five rats were randomly (random number table method) selected to establish the TBI model. After anaesthetizing with an intraperitoneal injection of sodium pentobarbital (50 mg/kg), the head was fixed on a stereotactic frame. Rectal temperature was measured and maintained at 37–37.5 °C by a heating pad. Following skull cutting with scissors, a craniotomy of 6 mm was made, centred around the coronal suture and 2.5 mm lateral to the sagittal suture. The impact velocity was set at 5.0 m/s, with a dwell time of 100 ms and a deformation depth of 2.5 mm. Afterwards, a severe TBI model was established. After emergence from anaesthesia, the rats were transferred to cages. Rats were provided with food and water ad libitum and evaluated daily during postoperative care via a physical examination and documentation of their condition. In the sham group, rats were subjected only to anaesthesia and scalp incision (with no craniotomy).

After TBI, the rats were randomized to the TBI, TBI + GLP-1(7–36)-L and TBI + GLP-1(7–36)-H groups (*n* = 14–15 per group). GLP-1(7–36) was purchased from MCE (cat. no. HY-P0054A, CAS: 1119517-19-9, China). GLP-1(7–36) intervention was conducted. Immediately after TBI, one group of rats received a low dose of GLP-1(7–36) (1 nmol in 5 µl i.c.v., once a day, for 30 days), and the TBI + GLP-1(7–36)-H group of rats received a high dose of GLP-1(7–36) (10 nmol in 5 µl i.c.v., once a day, for 30 days). Sham rats were injected with 5 µl saline only. One rat died during the modelling. Hence, rats were divided into four groups, namely, Sham, TBI, GLP-1(7–36)-L and GLP-1(7–36)-H.

### Modified neurological severity score (mNSS) score

To evaluate neurological functional outcomes, the mNSS test was performed at 24 h, 7 d and 30 d after TBI. The mNSS ranges from 0 to 18, and the mNSS includes the assessment of motor, sensory, balance, and reflex functions.

### Forelimb placement test and rotarod performance test

The forelimb placement test and rotarod performance test were used to assess the rat’s responsiveness to vibrissae stimulation and motor coordination and balance. Behavioural testing began 30 min before TBI and at 24 h, 3 d, 7 d and 30 d after TBI. According to a previous research method [26], rats were held, and their left vibrissae were stroked along the edge of a platform. Rats were considered to have a normal response if they put the forelimb ipsilateral to the stimulated vibrissae promptly onto the countertop. Ten measurements were obtained for each side in each rat at an interval of 5 min. The percent of appropriate forelimb in response to the vibrissae stimulation was calculated.

The rotating rod test was carried out according to previously published reports [26, 27]. Rats were placed on an accelerating rotarod apparatus initially rotating at 5 r.p.m. The rotational velocity gradually increased to 30 r.p.m. The duration until the mice fell from the rod was measured. If a mouse exceeded the 2-minute trial duration, a time of 120 s was recorded. Every measurement was carried out three times at an interval of 15 min by two independent researchers.

### Morris water maze (MWM) test

Learning, memory, and visual functions were evaluated through the MWM test 1–5 days and 26–30 days after TBI. The tests were carried out as in previous studies [24, 25, 27]. In brief, the MWM device consists of a pool (45 cm in height and 180 cm in diameter) and a movable platform (12 cm in diameter). Before initiation of the positioning navigation test, the rats were allowed to swim freely in the pool for 5 min. Each rat was placed into the water and randomly started from the other three quadrants not containing the hidden platform. All rats were allowed to find the platform within 90 s and stay on the platform for 15 s. If the rat failed to find the platform within 90 s, it was guided to the platform and allowed to stay for 15 s. On the 5th day, the platform was removed, and the time spent in the platform quadrant (within 90 s) was assessed. Three independent experiments were carried out (intertrial interval, 60 min). The behavioural data were automatically recorded and analysed with a video tracking system (HVS Image Software Ltd., Hampton, UK).

### Haematoxylin and eosin (H&E) staining

After behavioural tests, rats were anaesthetized with an intraperitoneal injection of sodium pentobarbital (50 mg/kg), perfused with paraformaldehyde and euthanized by decapitation. Then, hemispheres were rapidly extracted. The brain tissue samples were obtained 3 d following TBI and then subjected to HE staining. The brain tissues were immersed in 5% neutral buffered formalin for 7 d and postfixed in formalin. Then, the samples were sliced into 4-µm sections and stained with haematoxylin for 2 min and eosin for 30 s. Examination was performed in the hippocampal CA region with an Olympus microscope at magnification 200× for observation.

### Brain oedema detection

The wet/dry method was applied to detect the absolute brain water content. At 24 h, 7 d and 30 d after TBI, the cerebral cortex was harvested, and the wet weight (WW) was determined. Dry weight (DW) was obtained after drying tissues at 100 °C for 24 h. Cerebral oedema was calculated as (WW-DW)/WW× 100%.

### H_2_O_2_ detection

To measure H_2_O_2_ levels, we used an H_2_O_2_ detection ELISA kit (Molecular Probes, Monza, Italy). Hippocampal tissues were harvested at 24 h, 3 d, 7 d and 30 days after TBI. Fluorescence was determined with 550 nm excitation and 590 nm emission. The H2O2 content was calculated from a standard H_2_O_2_ curve [28].

### ROS analysis

ROS generation was determined using the stain 2,7-dichlorofluorescein diacetate (DCFH-DA; Sigma‒Aldrich, USA) in hippocampal tissues harvested from the animals at 48 h after TBI. Slices were preincubated with 10 µM DCFH-DA for 30 min. Then, the fluorescence of DCF was measured by laser confocal microscopy (Olympus Corporation, Tokyo, Japan).

### Antioxidant capacity evaluation

To assess the antioxidant capacity of hippocampal neuronal cells, the activities of CAT and SOD and the levels of reduced glutathione (GSH) were assessed at 48 h following the induction of experimental TBI. The Catalase Assay Kit (Cayman Chemical, USA) was used to assess the activity of CAT. The activity of SOD was determined by a superoxide dismutase activity assay kit (Abcam, Tokyo, Japan) according to the manufacturer’s instructions. Reduced GSH levels were determined using the GSH Assay kit (Cayman Chemical, USA) according to the kit instructions.

### Reverse-transcription quantitative PCR (RT-qPCR)

TRIzol® reagent (Invitrogen; Thermo Fisher Scientific, Inc.) was used to extract total RNA from hippocampal tissues. cDNA was prepared using a cDNA synthesis kit (PrimeScript™ RT reagent kit, Takara Bio, Inc., China) at 48 h after TBI. The reaction conditions were set at 37 °C for 15 min, followed by 5 s at 85 °C and then maintenance at 4 °C. PCR analyses were performed with TB Green™ Premix Ex Taq™ II (cat. no. RR820A; Takara Bio, Inc.). Each 20-µl PCR mix contained TB Green® Premix Ex Taq™ II, 2 µl template DNA, 0.8 µl forwards primer (10 µM), 0.8 µl reverse primer (10 µM) and 6.4 µl ddH2O. The thermal cycling step was 40 cycles at 94 °C for 60 s and 40 cycles at 95 °C for 18 s and 60 °C for 1 min. The final extension step was at 72 °C for 2.5 min. Measurements were made in triplicate. The primer sequences are detailed in Table 1. β-Actin was used as an internal reference gene, and the relative gene expression was calculated by the 2–(ΔΔCT) method [29].

**Table 1:** Real-time PCR primer sequences

Gene name		Primer sequence
Caspase‑3	forward primer	TACTCTACCGCACCCGGTTA
	Reverse primer	CGCGTACAGTTTCAGCATGG
Bax	forward primer	GTTGCCCTCTTCTACTTTGC
	reverse primer	ATGGTCACTGTCTGCCATG
Bcl-2	forward primer	GGCATCTTCTCCTTCCAGC
	reverse primer	TCCCAGCCTCCGTTATCC
ERK5	forward primer	CTGACGATGAGCCTGATTGC
	reverse primer	TGGACACACAGGCTCACTAG
CREB	forward primer	CTCGCTAACAATCGTACCGATG
	reverse primer	TCTTGCTGCTTCCCTGTTCTTC
β-actin	forward primer	AGCCATGTACGTAGCCATCC
	reverse primer	ACCCTCATAGATGGGCACAG

### Western blotting

The hippocampal tissues of rats were examined for the expression of proteins 48 h post TBI. Tissues were lysed in RIPA lysis buffer (Thermo Fisher Scientific, Inc.). The protein concentration was determined by a bicinchoninic acid (BCA) assay (BCA Protein Assay Kit; OriGene). Protein fractions were isolated in SDS-polyacrylamide gel electrophoresis (SDS‒PAGE) sample buffer, resolved by 12% SDS/PAGE and transferred to a PVDF membrane (Bio-Rad Laboratories, Inc.). Blocking was performed in 5% milk in TBS for 1.5 h at room temperature. Incubation with primary antibody was carried out overnight at 4 °C. The primary antibodies were as follows: ERK5 (cat. no. ab40809; 1:1,000; RRID:AB_732214; rabbit monoclonal; Abcam), p-ERK5 (cat. no. 3371; 1:1,000; AB_2140424; rabbit polyclonal; Cell Signaling Technology), CREB (cat. no. ab32515; 1:1,000; RRID:AB_2292301 rabbit monoclonal; Abcam), p-CREB (cat. no. ab32096; 1:1,000; RRID:AB_731734; rabbit monoclonal; Abcam), Bax (cat. no. ab32503; 1:1,000; RRID:AB_725631; rabbit monoclonal; Abcam), Bcl-2 (cat. no. ab196495; 1:1,000; RRID:AB_2924862; rabbit polyclonal; Abcam), CC-3 (cat. no. NB100-56113; 1:1,000; RRID: AB_3073989; rabbit polyclonal; Novus Biologicals) and β-actin (cat. no. ab8227; 1:1000; RRID:AB_2305186; rabbit polyclonal; Abcam). Secondary antibody (cat. no. ab216773; 1:10,000, RRID:AB_2925189; Abcam) incubation was performed at room temperature for 2 h. Enhanced chemiluminescence (ECL) detection was performed using ECL reagent (Bio-Rad Laboratories, Inc.). ImageJ (Image Lab 4.1; National Institutes of Health) was used to quantify the bands.

### Cell immunofluorescence

In our immunofluorescence experiments, 12-µm frozen sections were prepared and blocked for 1 h in blocking medium supplemented with 10% goat serum (cat. no. ab7481; RRID:AB_2716553; Abcam). The sections were incubated overnight with the primary antibodies p-ERK5 (cat. no. sc-135,760; 1:1,000; AB_2250338; mouse polyclonal; Santa Cruz Biotechnology) and NeuN (cat. no. EPR12763; 1:500; RRID: AB_2532109; rabbit monoclonal; Abcam) at 4 °C overnight. The next day, the sections were then incubated with secondary antibodies. The secondary antibodies used included Alexa Fluor® 488 goat anti-mouse IgG secondary antibody (cat. no. ab150113; 1:1,000; RRID:AB_2576208; Abcam) and Alexa Fluor® 647 donkey anti-rabbit IgG (H + L) secondary antibodies. Nuclei were counterstained with DAPI for 10 min. Fluorescence images were visualized with 400-fold magnification of an Olympus F1000 laser scanning confocal fluorescence microscope. The fluorescence intensity of hippocampal neurons was analysed with MATLAB software (MathWorks). For in vitro experiments, primary hippocampal neuronal cultures were generated (see below). Neurons were washed once with PBS and seeded in 6-well plates at 1-5 × 10^5^ cells/ml. The plates were fixed with 4% paraformaldehyde for 10 min on ice and blocked for 1 h in blocking medium supplemented with 10% goat serum. The subsequent protocol was identical to that performed in brain slices.

### TUNEL staining

Apoptosis was determined by TUNEL staining thirty days after TBI or sham surgery using a TUNEL Apoptosis Assay Kit (cat. no. C1088, Beyotime Institute of Biotechnology) as described previously [24]. Injured hemispheres were embedded in wax blocks and cut into paraffin sections. The slices (4 μm) were dewaxed and rehydrated. Following treatment with 10 µg/ml Proteinase K working solution, slices were rinsed again in PBS. Sections were stained with green fluorescein-labelled dUTP solution. Then, the sections were counterstained with DAPI (Vector Laboratories, Inc.) for 5 min. The total number of cells was assessed by the blue (DAPI) nuclei count, and the TUNEL-positive apoptotic cells exhibiting green fluorescent granules were counted. For the quantitative fluorescence microscopy analysis, five random visual fields (magnification, x200) were selected to count the cells.

### Cell culture and in vitro model of TBI

Primary hippocampal neurons were cultivated as previously described [30]. Hippocampal neurons were isolated from hippocampal tissues extracted from 17-day-old SD rat embryos (*n* = 54). Pregnant rats were anaesthetized with a 4.5% rate of isoflurane during induction and 2.5% during the maintenance period. Foetal rats were anaesthetized by brief carbon dioxide exposure and sacrificed by decapitation. Hippocampal tissues were dissected from the hemisphere of rats and digested with 0.25% trypsin (Gibco; Thermo Fisher Scientific, Inc.). After centrifugation (1,500xg, 5 min, 4 °C), the samples were resuspended in medium (neurobasal medium supplemented with 2% B27, 1% Glutamax and 2 µl gentamicin). Neurons were seeded at 1-5 × 105/ml in 6-well plates. After incubation for 24 h, we added arabinoside (10 mg/l) to the culture to inhibit glial proliferation. One-half of the culture medium was changed every 3 days.

Cells were randomly allocated into the following groups in vitro: Sham, TBI, TBI + GLP-1 (7–36), TBI + GLP-1 (7–36) + XMD8-92 and TBI + XMD8-92. We adopted a widely used method to develop an in vitro TBI model [31]. In vitro TBI was induced using a well-established mechanical stretch injury model [32]. Briefly, primary hippocampal neurons were seeded at a density of 700,000 cells per well in 6-well plates prior to scratch exposure. Subsequently, monolayers of cells were manually scratched using 10-µl micropipette tips at 4-mm intervals. Sham cells were cultured without treatment.

### Cell treatment and cell viability assay

Neurons from the corresponding treatment groups were cultured in RPMI 1640 medium that contained 0.1 mM, 1 mM, 10 mM and 100 nM GLP-1(7–36), along with 10 µM XMD8-92 (ERK5 inhibitor, cat. no. S7525, CAS: 1234480-50-2, Selleck Chemicals) for 48 h in 6-well plates. Cell viability was then assessed as described previously [30]. Next, we selected the most effective concentration for further experiments.

### Statistical analysis

Analyses were performed using SPSS (version 23.0). Each experiment was performed in triplicate and repeated three different times. The results are reported as the means ± standard deviations (SD). The results of the mNSS and behavioural experiments were analysed using a two-way mixed model ANOVA with Sidak’s post hoc test. The differences between various treatments were analysed by one-way ANOVA with Tukey’s post hoc test. Two-tailed *p* < 0.05 was regarded as statistically significant.

## Results

### GLP-1 (7–36) improved neurological disorders and cognitive deficits after TBI

**Fig. 1:**
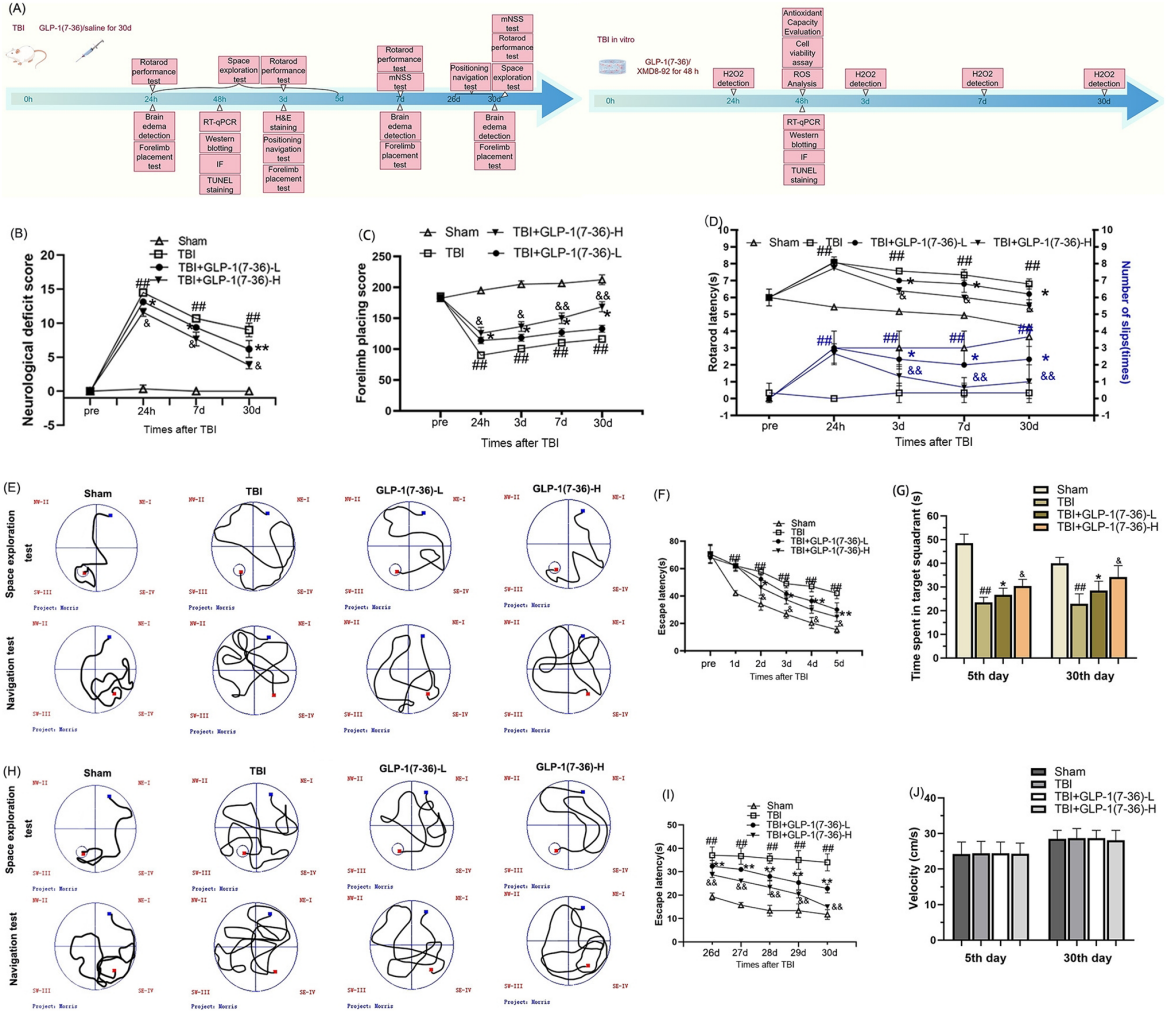
GLP-1 (7–36) improved neurologic deficits and cognitive dysfunction. **A** Experimental timeline. **B** Neurological functional outcomes were determined by the mNSS score. The rat’s responsiveness to vibrissae stimulation and motor coordination and balance were assessed by the **C** forelimb placement test and **D** rotarod performance test. The recovery of cognitive functions on days **E–J** 1–5 and **G–J** 26–30 was determined by the MWM test. Values are expressed as the means ± standard deviations. These behavioral assays were repeated in three to five biological and three technical replications. TBI, traumatic brain injury; GLP-1, glucagon-like peptide-1. (# P  < 0.05, ## P  < 0.01 compared with the Sham group; * P  < 0.05, ** P  < 0.01 compared with the TBI group; & P  < 0.05, && P  < 0.01 compared with the GLP-1 (7–36)-L group)

The neurological outcomes were assessed using the mNSS, rotarod test and forelimb placement test (Fig. 1). The TBI rat model exhibited significant neurological deficits at 24 h after TBI. Rats in the TBI + GLP-1(7–36)-L group showed lower mNSS scores over time from 24 h post TBI than rats in the TBI group. The ameliorative effect of GLP-1(7–36) was more pronounced in the high-dose group, suggesting the benefits of the higher doses (*P* < 0.05; Fig. 1B). These protective effects of GLP-1(7–36) were progressively enhanced over time (*P* < 0.05). After TBI, the forelimb placement accuracy of rats was impaired relative to that of the Sham group. Rats partially recovered forelimb-placing function over approximately 30 days after trauma in a dose-dependent manner (*P* < 0.05; Fig. 1B). TBI rats had apparent balance and coordination deficiencies relative to rats in the Sham group. Rats exhibited longer rotarod latencies after GLP-1(7–36)-L intervention. A high dose of GLP-1(7–36) resulted in a better performance in the rotarod performance test (*P* < 0.05; Fig. 1D).

Rats in the Sham group revealed a marked decrease in escape latencies from 1 d to 5 d after injury. In contrast, in the TBI group, there were significant differences in escape latencies compared with the Sham group. The escape latency tended to improve in GLP-1(7–36)-L rats and was more pronounced in GLP-1(7–36)-H rats (*P* < 0.05). In the space exploration experiment, we found a decrease in the time spent in the target quadrant in TBI rats (*P* < 0.01). Treatment with GLP-1(7–36) significantly extended learning and memory in TBI rats. The improvement was significantly greater in the high-dose group than in the low-dose group. The ameliorative effect of GLP-1(7–36) on spatial learning and memory deficiency became more significant at 26–30 days after TBI. There was no change in swim speed during the course of this experiment (*P* > 0.05; Fig. 1J).

### GLP-1(7–36) reduced cerebral oedema and neuropathological changes in tissues after TBI

**Fig. 2:**
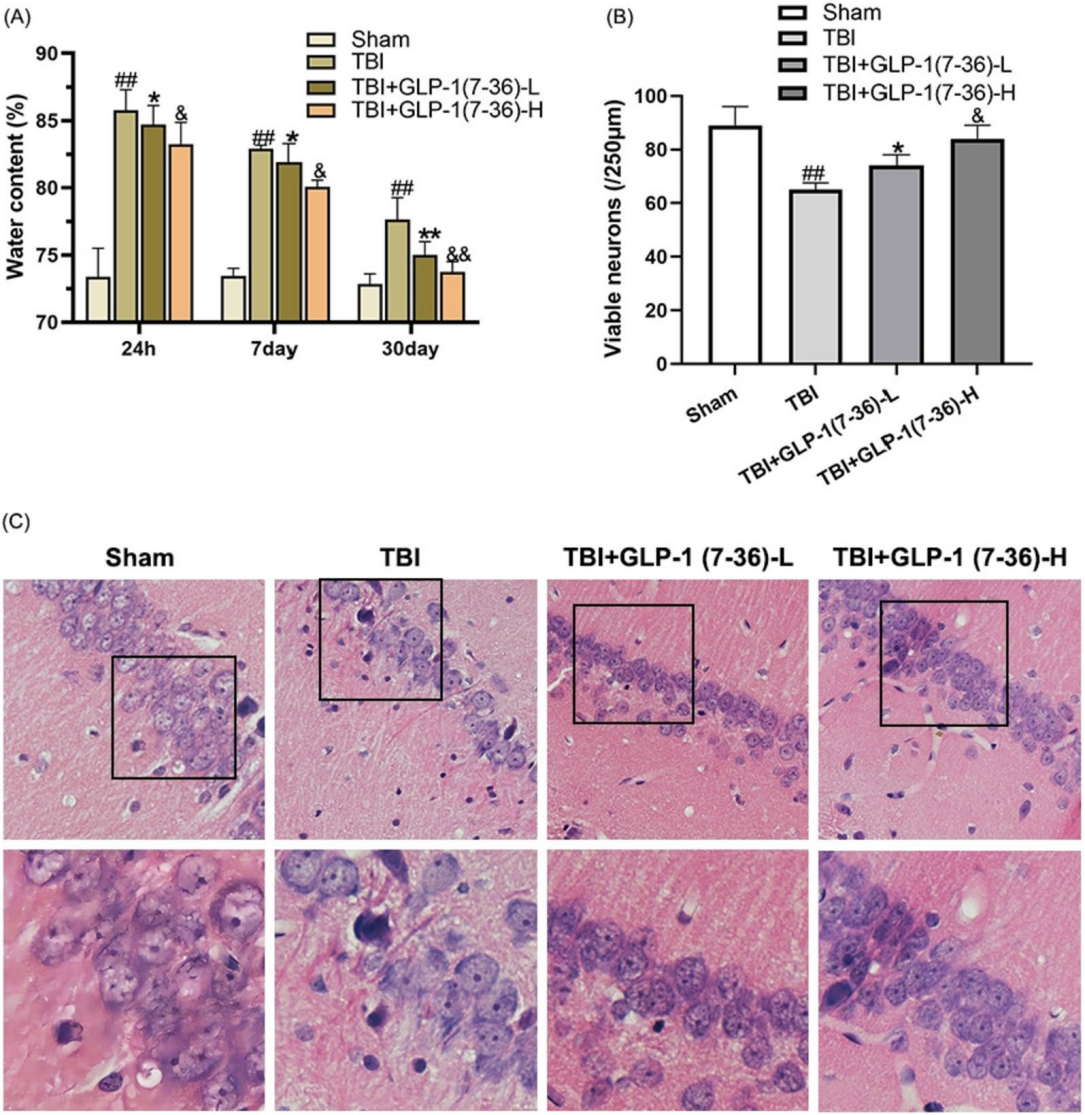
GLP-1(7–36) reduced brain oedema and pathological injury caused by TBI. **A** Brain water content was determined. **B** Quantification of the number of viable neurons per 250-lm length in each group. **C** H&E staining (scale bar, 100 μm). Values are expressed as the means ± standard deviations ( n  = 3 per group). TBI, traumatic brain injury; GLP-1, glucagon-like peptide-1. (# P  < 0.05, ## P  < 0.01 compared with the Sham group; * P  < 0.05, ** P  < 0.01 compared with the TBI group; & P  < 0.05, && P  < 0.01 compared with the GLP-1 (7–36)-L group)

The rats presented a significant amount of brain oedema at 24 h after TBI. The brain water content decreased at 7 d and 30 d. The water content was significantly increased compared with that of the sham group at the individual stages (*P* < 0.01). A lower dose of GLP-1(7–36) significantly reduced the water content of brain tissues, which was less with high-dose treatments (*P* < 0.05; Fig. 2A). H&E staining was used to assess pathological changes in hippocampal neurons (Fig. 2C). Neurons appear regular in shape in the Sham group, with round nuclei and granulated cytoplasm. Neurons appeared nuclear pyknotic, with condensed cytoplasm and fewer cells. A low dose of GLP-1(7–36) was able to effectively ameliorate the pathologic neural damage of TBI. Another observation worth mentioning is that more surviving neurons were observed after treatment with GLP-1(7–36), especially in the GLP-1(7–36)-H group (*P* < 0.05; Fig. 2B).

### GLP-1(7–36) decreased the contents of H2O2 and intracellular ROS and increased cellular antioxidant factors

**Fig. 3:**
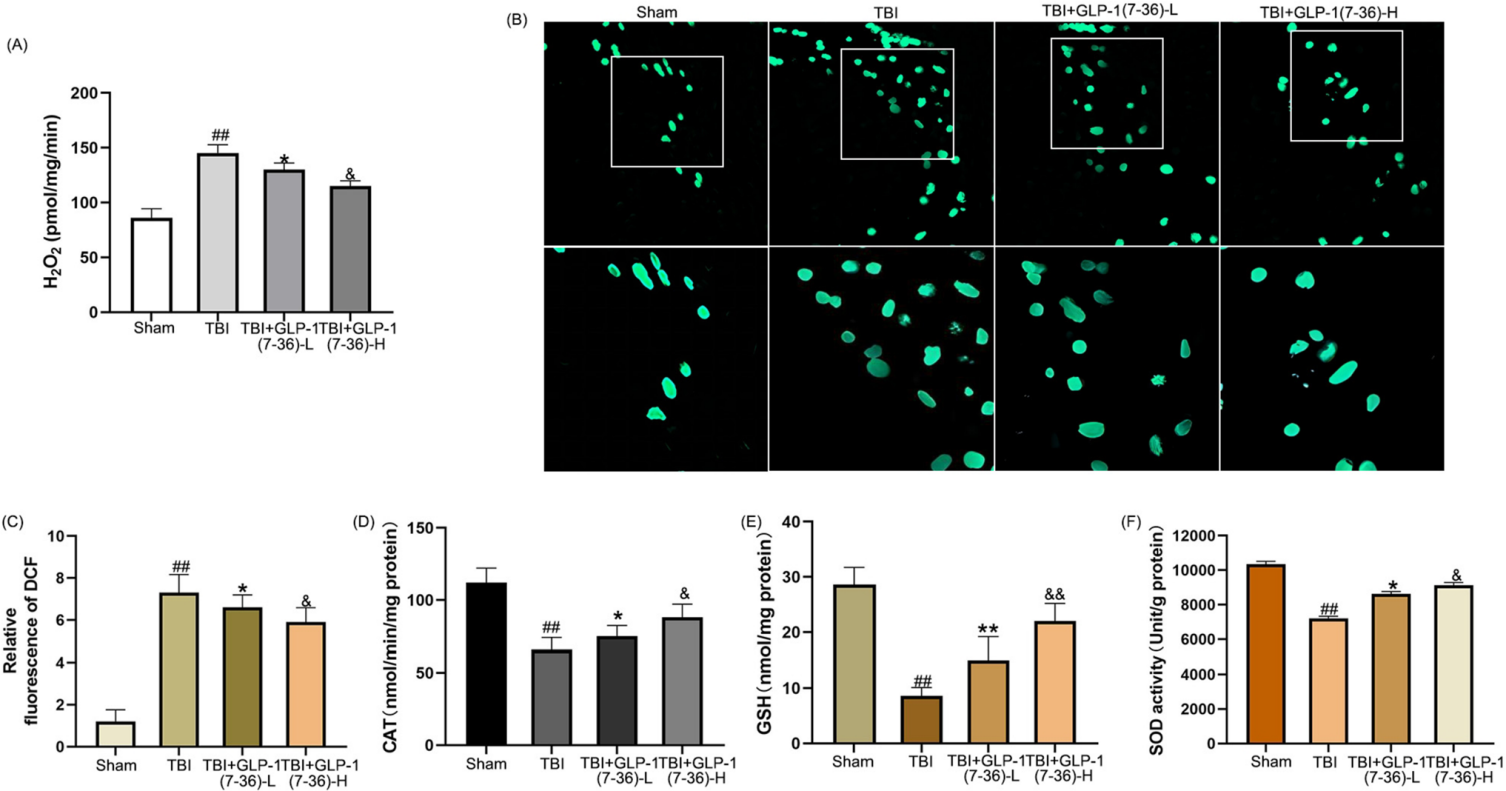
GLP-1(7–36) decreased the contents of H2O2 and intracellular ROS and increased cellular antioxidant factors. **A** The results show the release of H2O2 in the different groups. **B** Fluorescence images show DCF-stained hippocampal tissues. **C** Bar graphs showing the relative DCF fluorescence intensity in the hippocampal tissues. **D** CAT activity, **E** GSH levels and **F** SOD activity were detected to determine the antioxidant capacity. Values are expressed as the means ± standard deviations ( n  = 3 per group). TBI, traumatic brain injury; GLP-1, glucagon-like peptide-1; catalase, CAT; glutathione, GSH; superoxide dismutase, SOD. (# P  < 0.05, ## P  < 0.01 compared with the Sham group; * P  < 0.05, ** P  < 0.01 compared with the TBI group; & P  < 0.05, && P  < 0.01 compared with the GLP-1 (7–36)-L group)

As described in Fig. 3, the generation of H2O2 and ROS was markedly upregulated compared with that in the Sham group (*P* < 0.01), and H2O2 and ROS levels were markedly downregulated in the GLP-1(7–36)-L group and GLP-1(7–36)-H group (*P* < 0.05). The antioxidant effect was dose-dependent (*P* < 0.05). As shown in Fig. 3D and F, there was a drop in cellular antioxidant factors in TBI rats. The CAT and GSH levels and SOD activity were markedly upregulated in the GLP-1(7–36)-L group and GLP-1(7–36)-H group. Again, this effect was dose-dependent (*P* < 0.05).

### GLP-1(7–36) promoted the activation of the ERK5/CREB signalling pathways

**Fig. 4:**
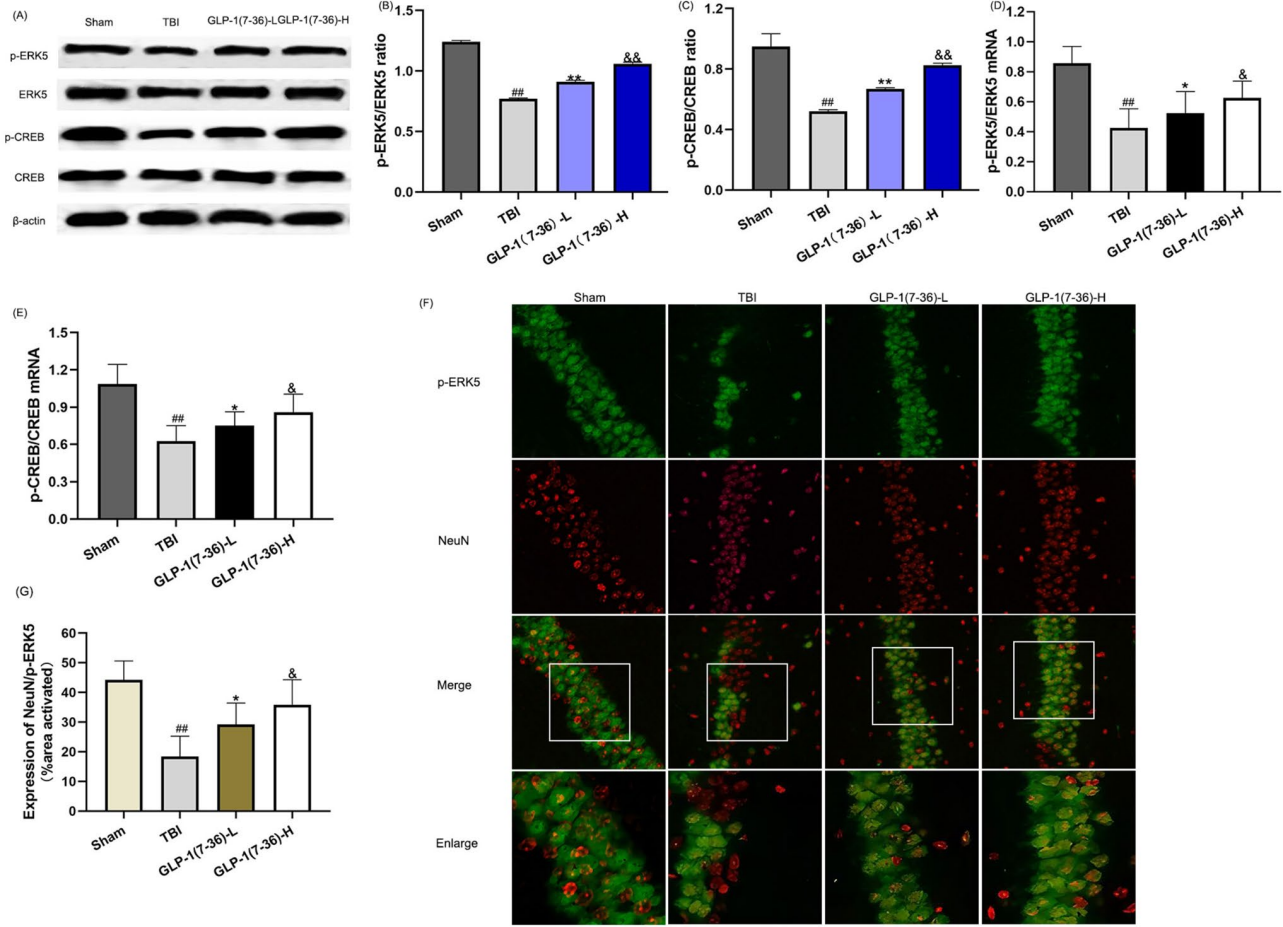
GLP-1(7–36) promoted the activation of the ERK5/CREB signalling pathways. **A** Representative western blots of proteins examined. The expression of (**B** and **D**) p-ERK5 and (**C** and **E**) CREB was determined by western blot analysis and reverse transcription-quantitative PCR. β-Actin was used as an internal control. **F** Representative double immunofluorescence staining of p-ERK5-positive cells (green) and NeuN-positive cells (red) in brain sections is displayed. **G** Statistical analysis of the p-ERK5-positive cell numbers in the observed area. Values are expressed as the means ± standard deviations ( n  = 3 per group). TBI, traumatic brain injury; GLP-1, glucagon-like peptide-1; ERK5, extracellular signal-regulated kinase; CREB, cAMP response element binding. (# P  < 0.05, ## P  < 0.01 compared with the Sham group; * P  < 0.05, ** P  < 0.01 compared with the TBI group; & P  < 0.05, && P  < 0.01 compared with the GLP-1 (7–36)-L group). (Color figure online)

As shown in Fig. 4, the western blot results showed that the ERK5/CREB pathway was inhibited in the TBI group compared with the sham group (*P* < 0.01). In addition, the PCR experiment exhibited the same trends (*P* < 0.01). There was an increase in p-ERK5 and p-CREB at the transcriptional or posttranscriptional level when rats were treated with GLP-1(7–36) (*P* < 0.05; Fig. 4B and E). Immunofluorescence images are shown in Fig. 4F. The fluorescence intensity of p-ERK5 was significantly decreased in the TBI group compared with the sham group (*P* < 0.01). GLP-1 (7–36) promoted more p-ERK5 expression, especially in the high-dose group (*P* < 0.05).

### GLP-1(7–36) suppressed apoptosis caused by TBI

**Fig. 5:**
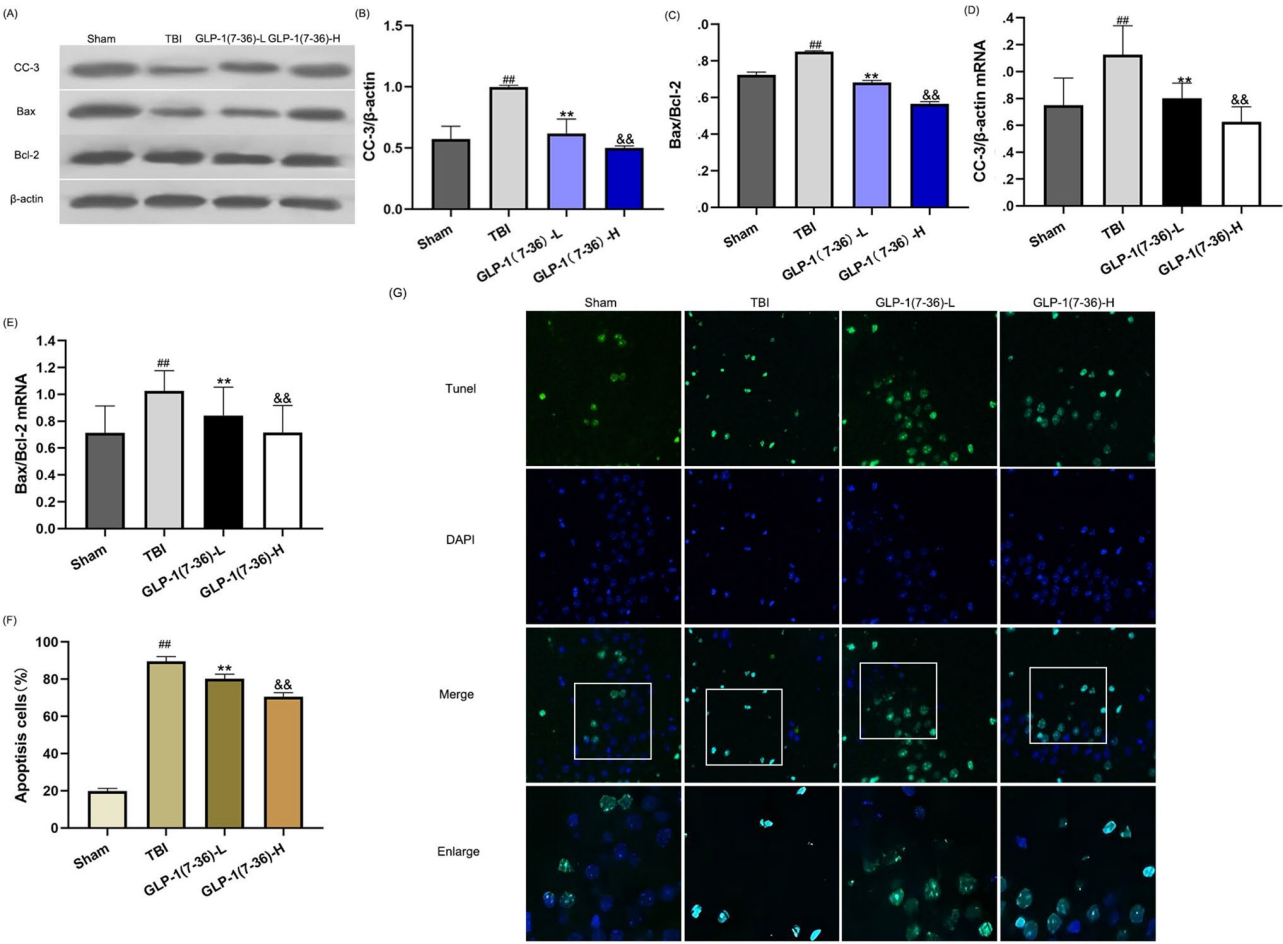
GLP-1(7–36) suppressed apoptosis caused by TBI. **A** Representative western blots of proteins examined. The expression of (**B** and **D**) CC-3 and (**C** and **E**) Bax/Bcl-2 was determined by western blot analysis and reverse transcription-quantitative PCR. β-Actin was used as an internal control. (**F**) Representative confocal images (scale bar, 50 μm) show neuronal apoptosis in hippocampal tissues based on TUNEL (green) and DAPI (blue) staining. (**G**) Bar graph shows the relative percentage of apoptotic neuronal cells in hippocampal tissues based on TUNEL staining. Values are expressed as the means ± standard deviations ( n  = 3 per group). TBI, traumatic brain injury; GLP-1, glucagon-like peptide-1; CC-3, cleaved caspase-3; Bcl-2, B-cell lymphoma-2. (# P  < 0.05, ## P  < 0.01 compared with the Sham group; * P  < 0.05, ** P  < 0.01 compared with the TBI group; & P  < 0.05, && P  < 0.01 compared with the GLP-1 (7–36)-L group). (Color figure online)

TBI significantly increased CC-3 and Bax/Bcl-2 compared with those in the Sham group (Fig. 5A and B; all *P* < 0.01). In comparison with rats in the TBI group, lower expression of CC-3 and Bax/Bcl-2 was observed in the GLP-1(7–36) group (*P* < 0.01), especially in the high-dose group (*P* < 0.01). Consistently, we found that the RNA levels of CC-3 and Bax/Bcl-2 were significantly reduced in rats treated with a low dose of GLP-1(7–36), and the high-dose group had a more obvious antiapoptotic effect (Fig. 5D and E). The same trend was observed in the PCR experiments. In the TUNEL assay (Fig. 5G), we found that hippocampal neurons in TBI rat brains survived less than those in Sham rat brains. Treatment with GLP-1(7–36) suppressed apoptosis compared to the TBI group, and the effect was more obvious in the high-dose group (*P* < 0.01).

### GLP-1 (7–36) significantly improved scratched cell viability

**Fig. 6:**
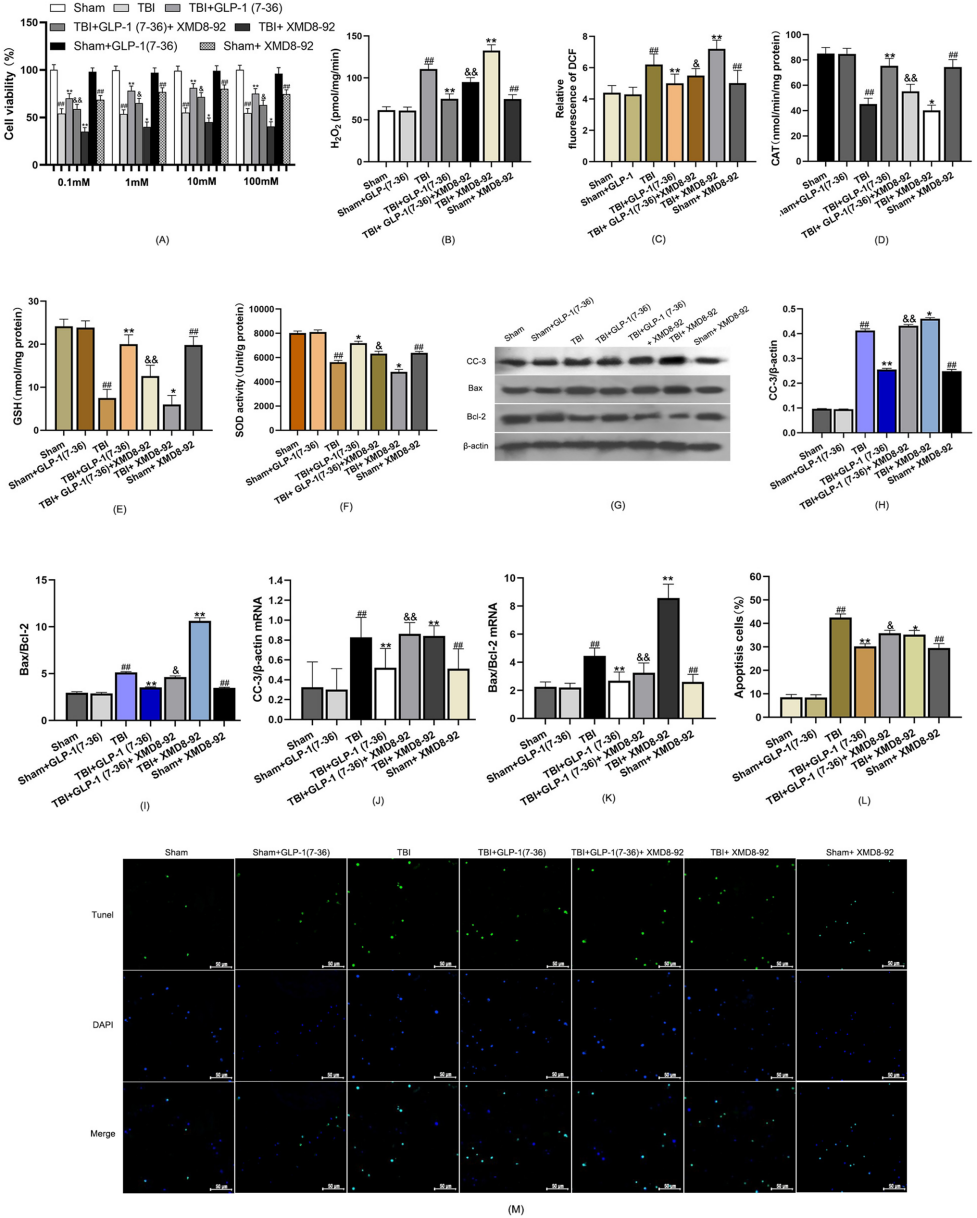
GLP-1 (7–36) increased cellular antioxidant capacity and inhibited apoptosis by activating ERK5/CREB signalling. **A** Cellular viability was detected using a CCK-8 assay. **B** The results show the release of H2O2 in the different groups. **C** Bar graphs showing the relative DCF fluorescence intensity in hippocampal neurons. **D** CAT activity, **E** GSH levels and **F** SOD activity were detected to determine the antioxidant capacity. **G** Representative western blots of proteins examined. The expression of (**H** and **J**) CC-3 and (I and K) Bax/Bcl-2 was determined by western blot analysis and reverse transcription-quantitative PCR. β-Actin was used as an internal control. **L** Bar graph shows the relative percentage of apoptotic neuronal cells in primary hippocampal neurons based on TUNEL staining. **M** Representative confocal images (scale bar, 50 μm) show neuronal apoptosis in primary hippocampal neurons based on TUNEL (green) and DAPI (blue) staining. Values are expressed as the means ± standard deviations. These experiments were repeated three times with five samples for each group. TBI, traumatic brain injury; GLP-1, glucagon-like peptide-1; catalase, CAT; glutathione, GSH; superoxide dismutase, SOD; CC-3, cleaved caspase-3; Bcl-2, B-cell lymphoma-2. (# P  < 0.05, ## P  < 0.01 compared with the Sham group; * P  < 0.05, ** P  < 0.01 compared with the TBI group; & P  < 0.05, && P  < 0.01 compared with the TBI + GLP-1 (7–36) group). (Color figure online)

Sham cells were used as controls for 100% cell viability (Fig. 6A). GLP-1 (7–36) did not alter cell viability in neurons without wounding. However, with the addition of XMD8-92, the viability of nonscrammed cells also decreased to a certain extent. Compared with the sham group, we found a low cell viability in the TBI group. Nevertheless, the viability of scratched neurons increased markedly after treatment with GLP-1 (7–36) (*P* < 0.05). Cell viability was decreased with the addition of XMD8-92, even after GLP-1(7–36) application. The concentration of 10 mM was used further for further experiments.

### GLP-1 (7–36) increased cellular antioxidant capacity by modulating the ERK5/CREB pathway

The intracellular H2O2 and ROS levels were significantly elevated after TBI relative to those in the sham group. There was a significant decrease in H2O2 and ROS generation with the addition of GLP-1 (7–36) (*P* < 0.01). In contrast, CAT and GSH levels and SOD activity decreased after TBI, and the cellular antioxidant capacity was enhanced in the group treated with GLP-1 (7–36) compared with the TBI group (Fig. 6B, C). The suppression of ERK5 by the addition of XMD8-92 markedly inhibited the antioxidative capabilities of neural cells after treatment with GLP-1 (7–36) (*P* < 0.05). This revealed that the protective effect of GLP-1 (7–36) was inhibited by ERK5/CREB.

### GLP-1(7–36) inhibits apoptosis by activating ERK5/CREB signalling

TBI significantly increased CC-3 and Bax/Bcl-2 expression compared with the Sham group (Fig. 6H and I). The levels of CC-3 and Bax/Bcl-2 were significantly decreased in hippocampal neurons following administration of GLP-1 (7–36) compared with the TBI group (both *P* < 0.01). Following inhibition of ERK5, the protein expression of CC-3 and Bax/Bcl-2 was significantly upregulated compared with that in the TBI + GLP-1 (7–36) group (*P* < 0.05), suggesting that the antiapoptotic effect of GLP-1 (7–36) is mediated through the ERK5/CREB pathway. These protein levels were consistent with the corresponding transcript levels (Fig. 6J, K). The TUNEL assay showed more TUNEL-positive cells in the TBI group than in the sham group. There was a significant decrease in TUNEL-positive cells in the TBI + GLP-1 (7–36) group after the addition of GLP-1 (7–36). Upon the addition of XMD8-92, the number of TUNEL-positive cells increased, even when GLP-1 (7–36) was added (Fig. 6L M).

## Discussion

When TBI occurs, neural cells exhibit varying degrees of nuclear pyknosis, nuclear fragmentation, and karyolysis. This will then result in neurological impairment and deficits in learning and memory, eventually leading to poor prognosis of TBI [33–37]. In the present study, GLP-1(7–36) was found to exert a role in the early phase after TBI in a dose-dependent manner. The neuroprotective effect was more pronounced in the later stage of TBI. After a traumatic event, substances released from damaged neurons led to increased BBB permeability and cytotoxicity, eventually resulting in cerebral oedema and increased intracranial pressure [38]. Studies have shown that SC administered liraglutide can alleviate inflammatory reactions and ameliorate ICH-induced cerebral oedema formation [39]. The inhibition of early inflammation could reduce the extent of brain tissue damage and early brain oedema [40]. There is no targeted therapy available against oedema formation, and symptomatic treatment is currently the main therapy for cerebral oedema [41–43]. In this study, GLP-1(7–36) was found to significantly decrease the water content of the cerebral cortex in a dose-dependent fashion. Meanwhile, administration of GLP-1(7–36) obviously improved the pathological injury of TBI-induced rats. One additional piece of evidence indicates the neuroprotective effect of GLP-1(7–36) [44] in AD. In parallel, the application of GLP-1 (7–36) depressed H2O2 generation in a concentration-dependent manner and minimized pathological neural injury. Therefore, we speculate that GLP-1 (7–36) may have the potential to be an agent for TBI rehabilitation.

GLP-1 (7–36) amide is an intestinal peptide released from L-cells upon glucose consumption and exerts antidiabetic effects [45–48]. Increased levels of GLP-1 (7–36) contribute to a decrease in biomarkers of oxidative stress (SOD, reduced glutathione, MDA, glutathione peroxidase, glutathione S transferase) [49–51]. Previously, in the study of the pathogenesis of atherosclerosis, GLP-1(7–36) was shown to reduce arachidonic acid-induced oxidative stress in platelets [52]. Another study revealed that GLP-1 (7–36) exerted antioxidative stress effects, increasing SOD, GSH-PX and eNOS. GLP-1 (7–36) decreased nerve function deficiency scores and significantly reduced infarction volume in an MCAO/R model [20]. It has been observed in a model of oxidative stress-induced toxicity in neuronal cells that administration of GLP-1(7–36) contributed to the amelioration of glutamate-induced oxidative stress and decreased intracellular reactive oxygen species [53]. This study fills a void with regard to the antioxidative effects of GLP-1 (7–36) in a TBI model. However, the underlying mechanism requires further study.

In addition to an increased oxidative stress response, apoptotic cell death is another pathophysiological aspect that affects neurological recovery after TBI [8, 54–60]. In the TBI model, trauma induced an increase in apoptosis, as evidenced by increased CC-3 and disruption of the balance between Bax and Bcl-2 [61–65]. Previous data have suggested that GLP-1(7–36) improved cell viability and cell survival in the HepG2 cell line, resulting in the downregulation of Bax mRNA [66]. Additionally, a previous in vivo study revealed that GLP-1(7–36) attenuated β1-42-induced neuronal cell death in murine hippocampal HT22 cells and improved pathological changes [53]. Our results also indicated that GLP-1(7–36) can downregulate apoptosis-related genes, namely, CC-3 and Bax. The TUNEL assay showed that GLP-1(7–36) inhibited apoptosis and contributed to the elevation of cell survival. With regard to the exact mechanisms through which GLP-1(7–36) inhibits apoptosis, it has previously been reported that GLP-1(7–36) regulates the autophagic process by activating ERK1/2 signalling pathways [53]. The ERK/CREB pathway is one of the three major MAPK pathways through phosphorylation of neighbouring proteins [67–70]. A previous study demonstrated that sustained ERK activation plays a key role in synaptic plasticity in the mammalian hippocampus [71]. The ERK-CREB cascade has been proven to be involved in neurodevelopmental processes, neuroplasticity, stress responses, and neurocognitive functions [72]. It is also known that the ERK-CREB cascade promotes cellular survival through two major factors: the ERK-dependent phosphorylation of prosurvival proteins and CREB-dependent transcription of prosurvival genes [73]. In the present work, obvious oxidative stress and apoptosis were observed in the in vitro and in vivo models of TBI, and the phosphorylation level of the ERK5/CREB pathway was simultaneously inhibited. When scratched primary neurons were treated with GLP-1(7–36), a significant decrease in apoptotic cells was observed. Long-term application of GLP-1(7–36) has been shown to reduce hippocampal neuronal damage by alleviating neuronal oxidative stress and neuronal apoptosis. The effect of GLP-1(7–36) in reducing CC-3, the Bax/Bcl-2 ratio and ROS generation was remarkably suppressed when ERK5 phosphorylation was inhibited by XMD8-92, indicating the essential role of ERK5/CREB in the antiapoptotic and antioxidative stress effects of GLP-1(7–36). Some researchers have proposed that GLP-1 can protect Müller cells from high glucose-induced damage by activating the ERK pathway, which attenuates oxidative stress [74]. A recent study suggested that GLP-1 significantly alleviated palmitic acid-induced neural death through ERK phosphorylation in pancreatic cells [75]. There were some limitations in this study. Additional pathways through which GLP-1 (7–36) plays a biological role, including GLP-1/cAMP and GLP-1/GIP/GCG, in the process of neural recovery after TBI were not studied in this current research. The prognosis of TBI is influenced by various factors, and additional in-depth investigations are necessary to determine the role of GLP-1(7–36) in other aspects of TBI pathogenesis. Additionally, the effective components of GLP-1(7–36) metabolites have yet to be determined. Therefore, further investigations are needed to obtain a comprehensive understanding of this matter.

## Conclusion

**Fig. 7:**
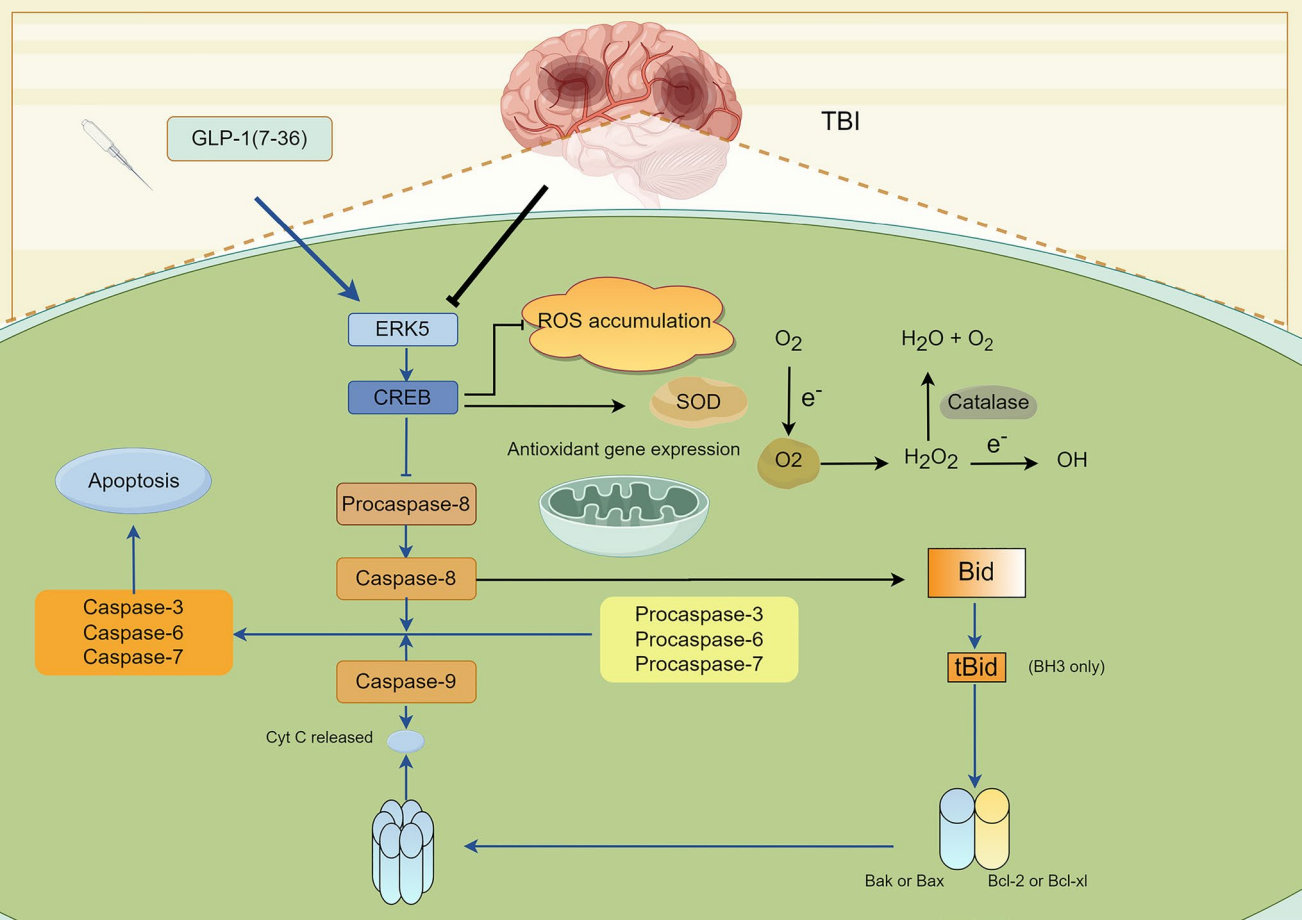
The mechanism’s diagram. GLP-1(7-36) mitigated neuronal damage following traumatic brain injury. The underlying mechanisms likely involve reducing oxidative stress by modulating the ERK5/CREB pathway, which includes decreasing ROS accumulation and enhancing the expression of antioxidant genes. Simultaneously, this pathway facilitates neuronal cell survival by inhibiting the activation of caspase family members and suppressing the proapoptotic Bcl-2 family members Bax or Bak

In conclusion, the present study demonstrated that GLP-1(7–36) alleviated neuronal damage after TBI, which was likely due to reducing oxidative stress and promoting neuronal survival via the ERK5/CREB pathway (Fig. 7). In addition, the results of this study further provide evidence that peptide hormone metabolites are promising treatments for neurological recovery post-TBI.

## Data Availability

The data used to support the fndings of this study areavailable from the corresponding author upon request.
